# Ready Method of Preparing Fomentations

**Published:** 1881-08-20

**Authors:** 


**A Ready Method of Preparing Fomentations.**—Dr. W. J. Fairfield, M.D., Chicago, says in Michigan Medical News: The most simple way that I have found for preparing fomentation cloths is as follows:

Take your flannel folded to the required thickness and size, dampened quite perceptibly with water, but not enough to drip, and place it between the folds of a large newspaper, having the edges of the paper lap well over the cloth so as to give no vent for steam. As it is now prepared lay it on the heated surface of the stove or register, and in a moment steam is generated from the under surface, and has permeated the whole cloth sufficiently to heat it the required temperature.

This method I was led to devise from necessity a few years ago, while in a sick room with no facility for heating water except in a quart cup. I have had occasion to use this way, and to instruct nurses to many times since, and I have always found in it a ready method which may be carried out in any sick room where there is a stove or register.

Never having seen any mention made of this, is my apology for now calling attention to it.

